# Supplementary material for: Vglut2-based glutamatergic signaling in central noradrenergic neurons is dispensable for normal breathing and chemosensory reflexes
Source: eLife. 2024 Sep 17;12:RP88673. doi: 10.7554/eLife.88673 (PMC11407767; doi:10.7554/eLife.88673)
Supplement: Supplementary file 1. [file elife-88673-supp1.docx]

**Supplementary File 1.** Vglut2 positive innervations from central noradrenergic neurons to the brain nuclei important in breathing control

| **Vglut2^+^ noradrenergic nuclei** | **Downstream target of Vglut2^+^ NA neurons** | **The role of the downstream target in breathing control** | **Reference** |
| --- | --- | --- | --- |
| **C1** | preBötzinger complex | Generate inspiratory rhythm | [Malheiros-Lima et al. (2018)](https://sciwheel.com/work/citation?ids=5607890&pre=&suf=&sa=0&dbf=1) |
|  | parafacial region (pFRG) | Regulate active expiration during chemosensory stimulation | [Malheiros-Lima et al. (2020)](https://sciwheel.com/work/citation?ids=8825854&pre=&suf=&sa=0&dbf=1) |
|  | Locus Coeruleus (LC) | Chemosensitive, regulate chemosensation | [Abbott et al. (2013)](https://sciwheel.com/work/citation?ids=1601019&pre=&suf=&sa=0&dbf=1); [Holloway et al. (2013)](https://sciwheel.com/work/citation?ids=4233129&pre=&suf=&sa=0&dbf=1) |
|  | A5 | Inhibitory drive for chemosensation and respiratory rhythm | [Abbott et al. (2013)](https://sciwheel.com/work/citation?ids=1601019&pre=&suf=&sa=0&dbf=1); [Malheiros-Lima et al. (2022)](https://sciwheel.com/work/citation?ids=12226944&pre=&suf=&sa=0&dbf=1) |
|  | A1, A2 | Modulate chemosensation, stabilize respiratory rhythm | [Abbott et al. (2013)](https://sciwheel.com/work/citation?ids=1601019&pre=&suf=&sa=0&dbf=1); [Holloway et al. (2013)](https://sciwheel.com/work/citation?ids=4233129&pre=&suf=&sa=0&dbf=1) |
|  | Lateral parabrachial nucleus (PBN) | Regulate chemosensation and arousal | [Abbott et al. (2013)](https://sciwheel.com/work/citation?ids=1601019&pre=&suf=&sa=0&dbf=1) |
|  | Medullary raphe | Regulate chemosensation and expiration | [Abbott et al. (2013)](https://sciwheel.com/work/citation?ids=1601019&pre=&suf=&sa=0&dbf=1) |
|  | Nucleus of the solitary tract (NTS) | Receive input from the carotid bodies (a key center for O_2_ chemosensation) and other cardiopulmonary afferents | [Abbott et al. (2013)](https://sciwheel.com/work/citation?ids=1601019&pre=&suf=&sa=0&dbf=1) |
|  | Dorsal motor nucleus of the vagus | Control respiration rate, regulate airway tone and defense | [Abbott et al. (2013)](https://sciwheel.com/work/citation?ids=1601019&pre=&suf=&sa=0&dbf=1) |
|  | Dorsomedial hypothalamus (DMH)  lateral hypothalamic area (LHA) | Modulate baseline respiration, chemosensation, arousal | [Abbott et al. (2013)](https://sciwheel.com/work/citation?ids=1601019&pre=&suf=&sa=0&dbf=1) |
|  | Paraventricular thalamic nucleus (PVT) | Regulate arousal | [Abbott et al. (2013)](https://sciwheel.com/work/citation?ids=1601019&pre=&suf=&sa=0&dbf=1) |
|  | Paraventricular nucleus  (PVN) | Modulate baseline respiration, respiratory response to hypoxia | [Abbott et al. (2013)](https://sciwheel.com/work/citation?ids=1601019&pre=&suf=&sa=0&dbf=1) |
| **Locus Coeruleus (LC)** | Lateral parabrachial nucleus | Regulate chemosensation and arousal | [Yang et al. (2021)](https://sciwheel.com/work/citation?ids=10322910&pre=&suf=&sa=0&dbf=1) |
